# Supplementary material for: Strain-specific epistasis shapes fitness landscapes of APOBEC3G antagonism by HIV-1 Vif proteins
Source: Sci Adv. 2026 Jul 23;12(30):eaed4872. doi: 10.1126/sciadv.aed4872 (PMC13394466; doi:10.1126/sciadv.aed4872)
Supplement: Supplementary file 1 — Figs. S1 to S5 Legends for data S1 to S6 [file sciadv.aed4872_sm.pdf]

Science Advances
AAAS

Supplementary Materials for

Strain-specific epistasis shapes fitness landscapes of APOBEC3G antagonism by HIV-1 Vif proteins

Caroline A. Langley *et al.*

Corresponding author: Caroline A. Langley, carolangley@gmail.com;
Michael Emerman, memerman@fredhutch.org

Sci. Adv. **12**, eaed4872 (2026)
DOI: 10.1126/sciadv.aed4872

The PDF file includes:

Figs. S1 to S5
Legends for data S1 to S6

Other Supplementary Material for this manuscript includes the following:

Data S1 to S6

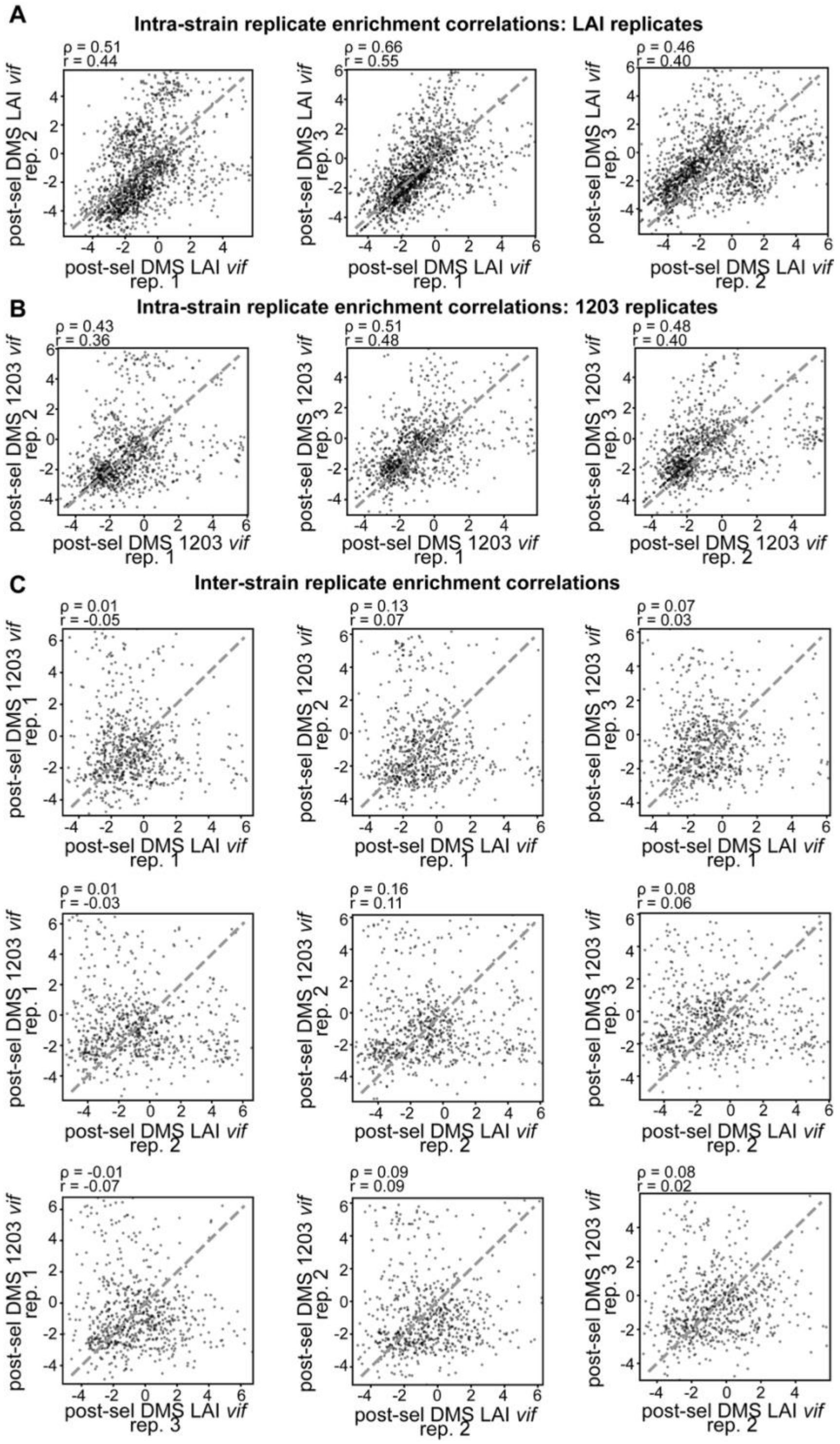

Fig. S1. Pairwise Spearman correlations of DMS enrichment scores across replicates and strains.
A. Intra-strain replicate correlations for HIV-1 LAI Vif. Scatter plots compare enrichment scores between biological replicates from the LAI DMS dataset. **B.** Intra-strain replicate correlations for HIV-1 1203 Vif. Scatter plots compare enrichment scores between biological replicates from the 1203 DMS dataset. **C.** Inter-strain correlations between LAI and 1203 Vif enrichment scores. Scatter plots compare enrichment scores across strains for all replicate pair combinations. In all panels, each point represents the enrichment score of a single Vif variant measured in the DMS assay, and Spearman (ρ) and Pearson (r) correlation coefficients are indicated.

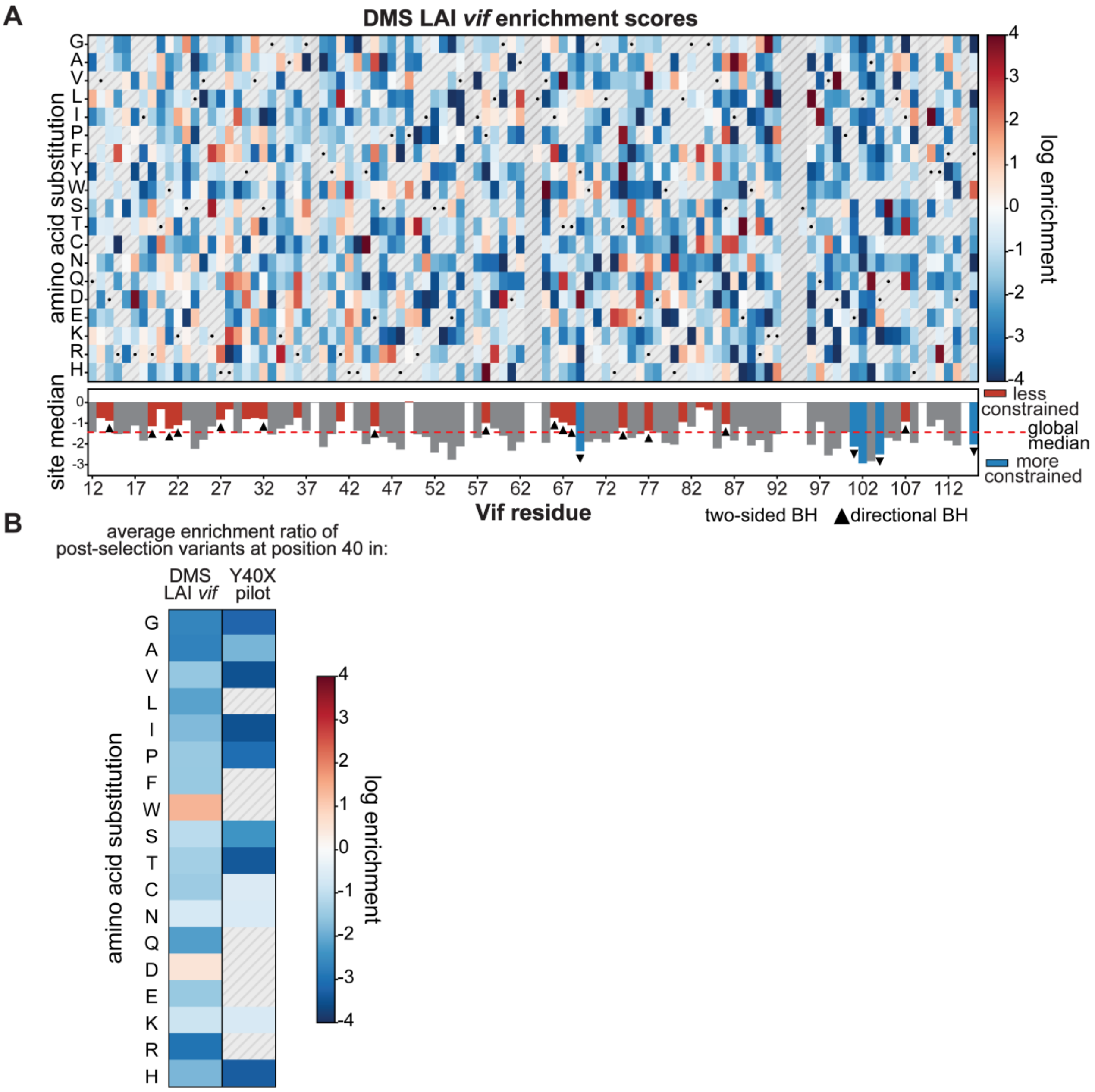

Fig. S2. DMS LAI *vif* enrichment following selection in SupT1-A3G cells

A. Top: Enrichment values reflect relative variant fitness; positive values (red) indicate retention or enhancement of A3G antagonism, whereas negative values (blue) indicate depletion after selection. Sites with insufficient variant coverage (<5 missense variants) are colored gray and hatched and excluded from statistical analyses. Additionally, sites 38, 56, 63–64, 93–95, 109, and 114 failed library quality control during synthesis and were excluded from analysis. Individual variants with insufficient representation are also shown in hatched gray. The wild-type amino acid at each site is denoted by a black dot. Bottom: Median log enrichment across all variants at each site. Significance was assessed using Wilcoxon signed-rank tests comparing variant enrichment values at each site to the library-wide median (dashed red line). Sites passing a Benjamini–Hochberg false discovery rate threshold ($q \leq 0.10$) in either a two-sided test (star) or a directionally consistent one-sided test (triangle) are highlighted as red bars (less constrained than average) or blue bars (more constrained than average). All other sites are shown as gray bar. **B.** Comparison of enrichment values for amino acid substitutions at Vif residue 40 between the full DMS LAI vif dataset and the targeted Y40X pilot dataset.

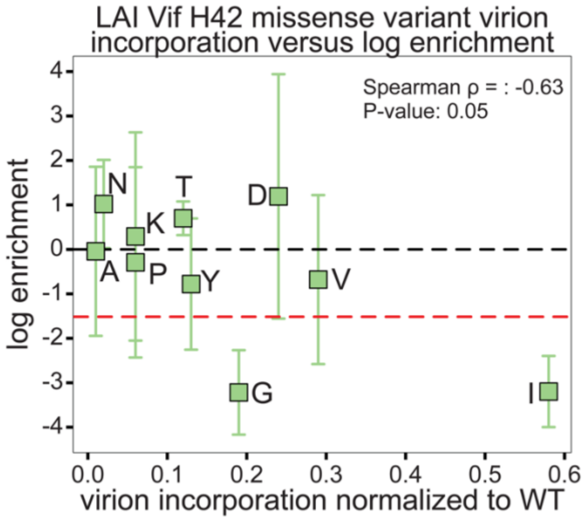

Fig. S3. LAI Vif H42 variant enrichment versus virion incorporation Plot comparing enrichment scores with virion incorporation normalized to the wild-type Vif level for LAI Vif H42 missense variants. Each point represents a single variant and is labeled by the substituted amino acid. Error bars indicate variability in enrichment across replicates. Lower virion incorporation was associated with higher enrichment across variants (Spearman ρ = −0.63, p = 0.05).

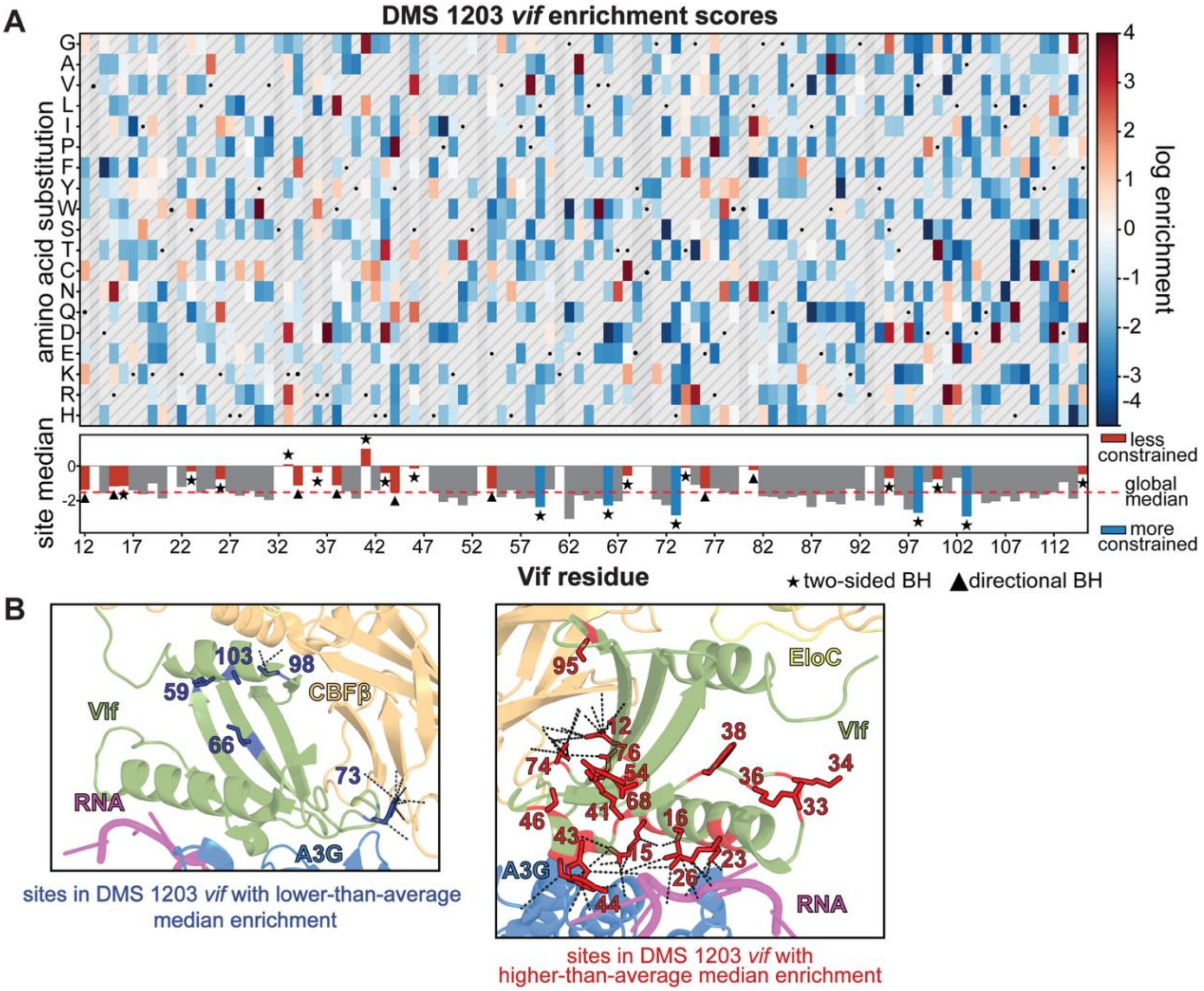

Fig. S4. DMS 1203 *vif* dataset heatmap and sites that are significantly more or less constrained than the library-wide average. A. Top: Heatmap showing the log enrichment ratios for all single missense substitutions across residues 12–115 of 1203 Vif following two rounds of selection in SupT1 cells expressing A3G. Enrichment values reflect relative variant fitness; positive values (red) indicate retention or enhancement of A3G antagonism, whereas negative values (blue) indicate depletion after selection. Sites with insufficient variant coverage (<5 missense variants) are colored gray and hatched and excluded from statistical analyses. Additionally, sites 21, 35, 45, 47, 53, and 93 failed library quality control during synthesis and were excluded from analysis. Individual variants with insufficient representation are also shown in gray and hatched. The wild-type amino acid at each site is denoted by a black dot. Bottom: Median log enrichment across all variants at each site. Significance was assessed using Wilcoxon signed-rank tests comparing variant enrichment values at each site to the library-wide median (dashed red line). Sites passing a Benjamini–Hochberg false discovery rate threshold ($q \leq 0.10$) in either a two-sided test (star) or a directionally consistent one-sided test (triangle) are highlighted as red bars (less constrained than average) or blue bars (more constrained than average). All other sites are shown as gray bars. B. Sites with significantly lower-than-average (left) and higher-than-average (right) median log enrichment scores (from Fig. 5B, bottom panel, blue and red bars) are mapped onto the LAI Vif–A3G–VCBC–RNA cryo-EM structure (PDB: 8CX0). Gray dashed lines indicate known protein–protein and protein–RNA interactions.

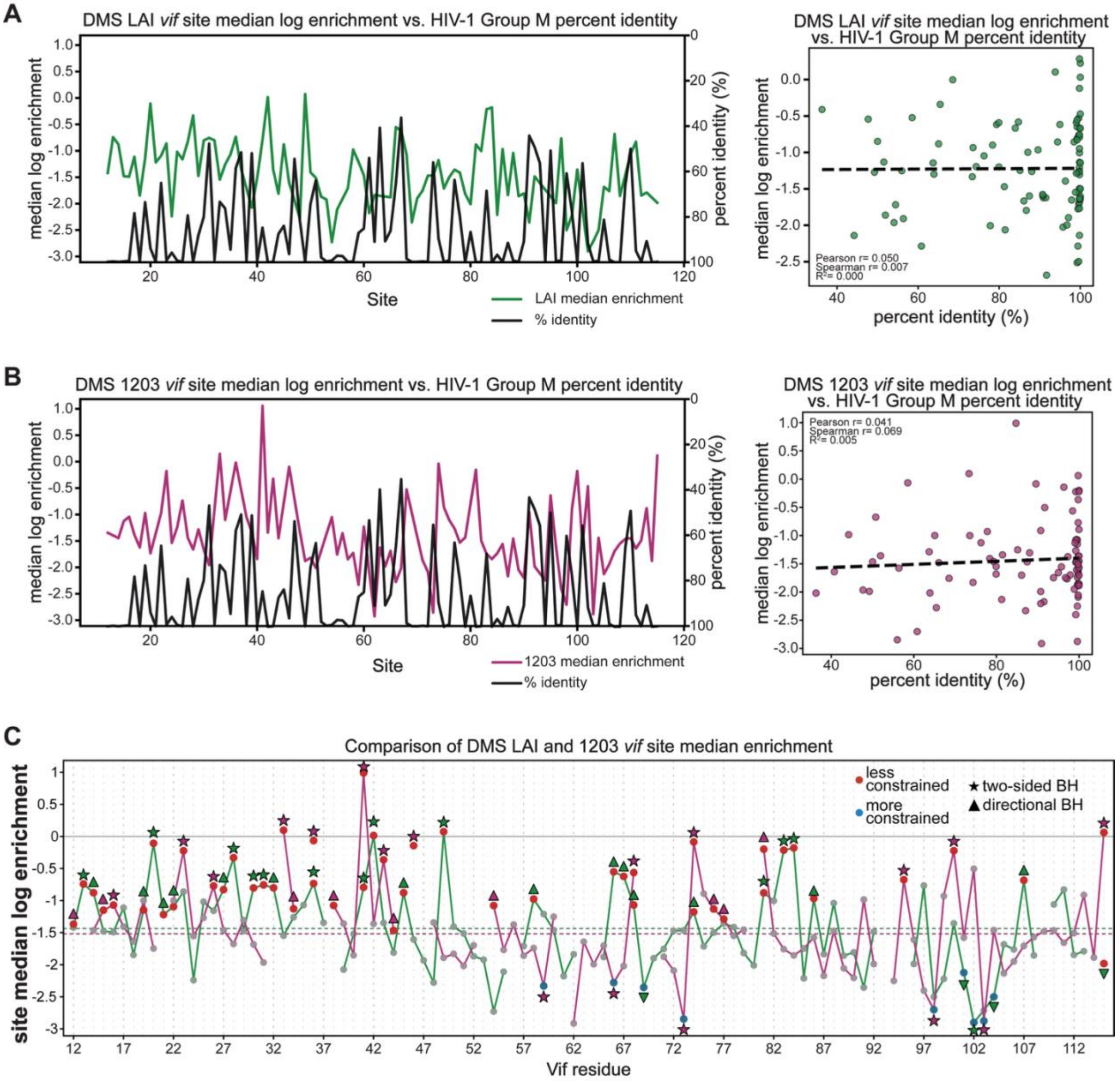

Fig. S5. Comparison of functional constraint (via DMS) and evolutionary conservation in HIV-1 Vif. **A.** Per-site median log enrichment values from DMS LAI *vif* plotted against natural sequence conservation across HIV-1 Group M sequences. Left panel: Median enrichment values for each Vif residue (sites 12–115; green line, left y-axis) shown alongside percent identity to the modal (consensus) amino acid at each site in a Group M alignment (black line, right y-axis). Right panel: Scatterplot comparing per-site median enrichment and consensus percent identity. Each point represents one Vif residue. Dashed black line indicates the linear regression fit; Pearson's r, Spearman's ρ, and coefficient of determination (R^2) are shown. **B.** Same as in A, but for DMS 1203 *vif* (purple line). **C.** Overlay of site-level median enrichment values for DMS LAI *vif* (green) and DMS 1203 *vif* (purple). Median enrichment values closer to 0 (solid gray line), or greater than 0, indicate greater mutational tolerance, whereas increasingly negative values indicate stronger functional constraint. To identify sites whose variant-level enrichment values significantly deviated from the library-wide median enrichment (dashed green line for LAI; dashed purple line for 1203), Wilcoxon signed-rank tests were performed comparing the distribution of variant enrichment values at each site to the corresponding library-wide median. Sites passing a Benjamini–Hochberg false discovery rate threshold ($q \leq 0.10$) in either a two-sided test (star) or a directionally consistent one-sided test (triangle) are highlighted in red (less constrained than the library-wide median) or blue (more constrained than the library-wide median). Sites not meeting significance thresholds are shown in gray.

Data S1. DMS LAI *vif* library QC and variant proportions

Tabulated summary showing site-level and variant-level QC thresholds, variant codons, the number of sites analyzed, and counts of passed and failed sites.

Data S2. DMS 1203 *vif* library QC and variant proportions

Tabulated summary showing site-level and variant-level QC thresholds, variant codons, the number of sites analyzed, and counts of passed and failed sites.

Data S3. Enrichment ratio metrics for DMS *vif* library variants
Comprehensive table reporting variant-level enrichment scores from the DMS *vif* experiment. For each mutation, codon-level and amino acid-level substitutions are shown alongside their observed pre- and post-selection counts and frequencies.

Data S4. Group M Vif Subtype Reference Alignment
Sequence alignment of representative Vif sequences from Group M viruses

Data S5. Clade B Vif Subtype Reference Alignment
Sequence alignment of representative Vif sequences from Clade B viruses

Data S6. Python code used for analysis of DMS sequencing data
